# Cell Encapsulation in Sub-mm Sized Gel Modules Using Replica Molding

**DOI:** 10.1371/journal.pone.0002258

**Published:** 2008-05-21

**Authors:** Alison P. McGuigan, Derek A. Bruzewicz, Ana Glavan, Manish Butte, George M. Whitesides

**Affiliations:** Harvard University, Cambridge, Massachusetts, United States of America; Dresden University of Technology, Germany

## Abstract

For many types of cells, behavior in two-dimensional (2D) culture differs from that in three-dimensional (3D) culture. Among biologists, 2D culture on treated plastic surfaces is currently the most popular method for cell culture. In 3D, no analogous standard method—one that is similarly convenient, flexible, and reproducible—exists. This paper describes a soft-lithographic method to encapsulate cells in 3D gel objects (modules) in a variety of simple shapes (cylinders, crosses, rectangular prisms) with lateral dimensions between 40 and 1000 μm, cell densities of 10^5^ – 10^8^ cells/cm^3^, and total volumes between 1×10^−7^ and 8×10^−4^ cm^3^. By varying (i) the initial density of cells at seeding, and (ii) the dimensions of the modules, the number of cells per module ranged from 1 to 2500 cells. Modules were formed from a range of standard biopolymers, including collagen, Matrigel™, and agarose, without the complex equipment often used in encapsulation. The small dimensions of the modules allowed rapid transport of nutrients by diffusion to cells at any location in the module, and therefore allowed generation of modules with cell densities near to those of dense tissues (10^8^ – 10^9^ cells/cm^3^). This modular method is based on soft lithography and requires little special equipment; the method is therefore accessible, flexible, and well suited to (i) understanding the behavior of cells in 3D environments at high densities of cells, as in dense tissues, and (ii) developing applications in tissue engineering.

## Introduction

Growing cells on rigid 2D substrates is a standard and convenient method for cell culture. For many types of cells, however, a rigid 2D environment does not accurately provide the mechanical and chemical cues experienced *in vivo*, and significant differences in behavior of cells therefore exist between 2D and 3D culture [1]–[4]. To study the behavior of cells in 3D environments, there is a need for 3D culture systems that (i) accurately mimic the environment of *in vivo* tissues; (ii) conveniently integrate with current tools of biology (such as optical microscopy and high-throughput screening); and (iii) provide flexibility similar to that of current 2D culture, in terms of the ability to control experimental variables, to study different cell types, and to ask a wide range of biological questions. Such systems for 3D culture would be particularly useful for studying 3D biological phenomena, such as the organization and pathology of tissues, or the metabolism and distribution of drugs. This paper describes an *in vitro*, 3D system based on soft lithography [5] that enables the culture of cells in biologically relevant environments at tissue-like cell densities (10^7^ – 10^9^ cells/cm^3^). This system is simple to use and versatile, and it encapsulates cells in a highly reproducible form that is compatible with standard biological tools for characterization and high-throughput screening.

Existing methods for 3D cell culture include (i) culturing explants of native tissues or organs [6], [7], (ii) culturing 3D aggregates of cells [8]–[11], and (iii) encapsulating cells in biopolymers or other matrixes to form an “engineered tissue” [12]–[15]. By isolating actual tissue, the culture of explants provides an *in vitro* system that very accurately provides the *in vivo* 3D microenvironment of tissues, but this approach has limited use for high-throughput studies, since variation among samples of tissue from different sources makes comparisons difficult. For each type of tissue, the choice of culture medium and procedure for dissection must be optimized, and samples live for only two to three days. Furthermore, control over experimental variables, such as the presence of growth factors, is limited.

Cultures of cellular aggregates mimic the tissue microenvironment less accurately than do explants, but systems based on aggregates offer greater reproducibility. Since hundreds of nearly indistinguishable aggregates can be generated simultaneously, they integrate more conveniently with high-throughput technology [9], [16]. Unfortunately, only some types of cells form cellular aggregates, and because the extracellular matrix of the aggregate is secreted by the component cells, little control over which matrix components are present is possible. Cellular aggregates also tend to agglomerate, and cells may die in regions where nutrients can no longer reach the cells by diffusion [17].

Engineered tissues, like cellular aggregates, mimic the native microenvironment less accurately than do tissue explants. Unlike aggregates, engineered systems can be highly reproducible in terms of (i) size and shape of the engineered tissue, (ii) the type of cells and source of the biomaterial scaffold/matrix used to build the engineered tissue, and (iii) the number of cells per unit volume enclosed. Engineered systems can also be more flexible than culture of aggregates, since a greater variety of cell types and matrix proteins can compose the (initial) microenvironment of the engineered system. Most methods of tissue engineering, however, are not standardized, and tend to focus on the production of one, relatively large (mm- to cm-scale) section of tissue. These large engineered tissues are not ideal systems for 3D cell culture because (i) large slabs do not easily integrate with high-throughput screens, and (ii) unlike natural tissues, engineered tissues lack a vasculature to supply blood or nutrients. Diffusion of nutrients and waste to and from the center of the engineered tissue therefore limits the maximum possible density of healthy cells in tissues with dimensions greater than ∼200 μm [18], [19].This limitation forces many experimental systems based on engineered tissues to operate at densities of cells far below those of tissues *in vivo*, and particularly affects the culture of tissues, such as liver, with high metabolic rates per cell. Some top-down approaches have been developed to create artificial matrixes based on scaffolds with channels for nutrient delivery to enable culture at high density of cells [20], [21]; the present work focuses on a miniaturization strategy to allow culture at high densities of cells and integration with standard imaging and high-throughput tools. A method to generate large numbers of indistinguishable 3D engineered tissues with dimensions below ∼200 μm and high densities of cells would provide an attractive 3D culture system for controlled studies of 3D cell biology.

McGuigan and Sefton previously proposed *modular* tissue engineering as a strategy to assemble vascularized “constructs” from sub-mm sized cell-containing modules [22]. Instead of seeding a preformed scaffold with cells, they encapsulated the desired type of cells in cylinders (2-mm long, 600-μm wide; collagen concentration of 3 mg/mL) of collagen-I gel, and then covered the outer surface of the modules with a confluent layer of endothelial cells (EC) [22] (see Supplemental Information Figure S1 for a schematic representation of this design). The loose packing of the modules ensured that no part of any module was more than ∼250 μm from an open channel at any time; therefore, when a syringe pump forced cell culture medium through the construct, the liquid permeated the network of channels, and nutrients reached all cells in the modules by diffusion.

In theory, modular tissue-engineered constructs are an attractive option for 3D cell culture. In addition to the general advantages offered by engineered systems, modular tissues possess three desirable qualities: (i) the ability to reach cell densities like those of native tissue without necrosis, (ii) easy integration with optical microscopy and high-throughput screening, and (iii) versatility in terms of the choices and combinations of cell types and matrix materials used. For gel modules with lateral dimensions below 200 μm (2× the maximum diffusion length of 100 μm), limitations on mass transport via diffusion into and out of the gel matrix would not prevent proliferation of cells to uniform densities (cells per unit volume) near those of *in vivo* tissues. The small size and reproducible fabrication of modular tissues would ensure that modules integrate conveniently with current biological tools. The flexibility of the fabrication method would allow (i) different modules to contain different types of gel matrix, (ii) single modules to contain controlled mixtures of types of cells, and (iii) modules that contain different types of cells to be mixed. Modular constructs could be assembled for a particular biological test and then disassembled for analysis of the cells in the different modules after the test. If desired, dissolution of the modules could release the individual living cells for separate analysis. Alternatively, experiments could be conducted on individual modules without assembling a modular tissue. The goal here was to demonstrate the advantages of modular tissue engineering as a 3D cell culture system.

Only a simple method for making modules will make this system accessible to investigators in the biology community. Current methods to make modules are limited to cylindrical shapes with diameter ≥500 μm [23], [24]. Modules with more complex shapes can be fabricated, but only with the use of specialized lithography equipment [25].The method presented here demonstrates (i) reproducible generation of tens to thousands of indistinguishable modules in parallel, with dimensions from below 100 μm to at least 1000 μm, using a number of biopolymers commonly used for cell encapsulation; (ii) modules with a range of cell densities, up to approximately the density of native tissue (10^8^ –10^9^ cells/cm^3^); (iii) viability and metabolic activity of the cells in the modules for at least one week; (iv) cell densities in the modules that can be controlled by varying the density of seeded cells and the dimensions of the modules and (v) use of the module system to observe differences in cell-cell interactions and function on a 2D surface versus within a 3D environment. By widening the scope of the currently available methods with a simple technique, we hope to encourage use of the modular approach in applications outside of tissue engineering, and to provide a simple tool for biological investigations of the behavior of cells in 3D at high density.

## Results

### Module fabrication technique

**Figure 1:**
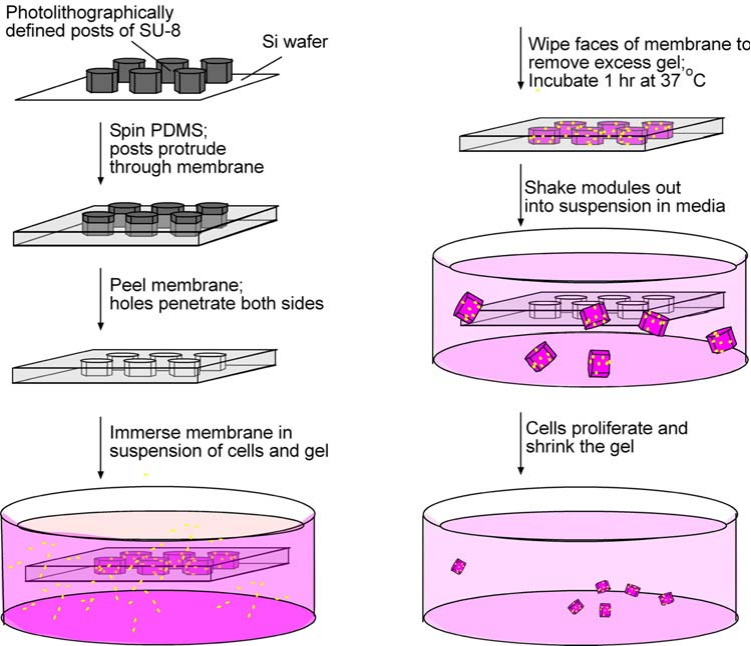
Schematic diagram of module fabrication.

We formed poly(dimethyl siloxane) (PDMS) membranes to serve as templates for gel structures (modules) using replica molding [5]. (Figure 1 is a schematic of process). We used photolithography of SU-8 photoresist on a silicon wafer to generate “master” posts with diameters from 40 to 1000 μm and heights from 100 to 1000 μm [5]. Spin-casting PDMS around the posts generated a membrane that bore an array of holes in a variety of shapes with precise dimensions (see Materials and Methods). These holes fully penetrated the membrane, and allowed the gel modules to be released easily after formation (see below). Depending on their size, between dozens and hundreds of modules could be conveniently formed at one time in the membrane. For example, we could fit over 900 holes of 1-mm diameter into one 7 cm×3 cm sheet. Both the PDMS membranes and the master posts were reusable at least dozens of times.

To prepare the membranes for treatment with the gel, we oxidized them in an air plasma to render their surfaces hydrophilic, placed them in jars filled with water, degassed the jars under house vacuum to remove air bubbles, and autoclaved the membranes in the jars to preserve the hydrophilicity and sterility of the PDMS. Keeping the surfaces of the membranes hydrophilic until the time of use both ensured that the biopolymer gel filled the holes in the membrane, and reduced formation of air bubbles in these holes.

Modules were formed by mixing NIH 3T3 cells (1×10^6^ cells per mL of neutralized collagen) with a solution of neutralized collagen (concentration 3 mg/mL, pH 7), and then loading this mixture of cells and gel into the holes (with diameters 40 – 1000 μm) in the PDMS membrane. The holes were loaded with the gel by submerging the entire hydrophilic membrane (3 cm by 3 – 7 cm) in a 1 – 5 mL suspension of cells in a solution of collagen in a Petri dish. Gently shaking the membrane using tweezers released any bubbles of air transiently trapped in the holes in the membrane. We scraped the flat faces of the membrane against the sterile edges of the Petri dish to remove excess gel, and then suspended the membrane in the air and incubated at 37 °C for 45 minutes to allow gelation of the collagen. After gelation, we immersed the membrane in cell culture medium and then agitated the membrane with tweezers for 10 – 15 minutes, until the gel modules separated from the holes in the membrane. Once free from the PDMS membrane, and suspended in cell culture medium, the cell-containing gel modules—with shapes of cylinders, crosses, and rectangular prisms—retained their shape without tearing or collapsing (the size of the module decreased within one day because of remodeling of the gel by cells—see below). Fabrication of the modules using the membranes required approximately 1 hour (not including the time necessary to produce the membranes). To prevent the modules from clumping together, we pipetted them up and down every two days, and changed the culture medium twice weekly.

### Characterization of cell viability and cell density within the modules

To show that cells encapsulated in the modules remained alive and metabolically active after seven days in culture, we used the Alamar Blue assay [26] and the Trypan Blue assay. For the Alamar Blue assay, we placed samples of 50 identical modules in separate wells of a 96-well plate. We added a set volume of cell culture medium to each well, and supplemented the medium with the Alamar Blue compound (resazurin). Living cells take up the blue compound and convert it to a pink metabolic product. The ratio of absorbance by the media of 570 nm light to absorbance of 600 nm light then serves as a readout of how much resazurin has been converted; metabolically active cells show an increase in absorbance of 570 nm light relative to absorbance of 600 nm light over time. The ratio of absorbance values at a given point in time thus gives a semi-quantitative indication of the total amount of metabolic activity in a sample of modules. Incubating 50 modules with resazurin for ∼2 h caused the solution to turn pink. This change in color indicated that cells were metabolically active in the modules. To demonstrate that cells in the various sizes of modules showed comparable metabolic activity, we dissolved the collagen matrix with trypsin and counted the cells; we then calculated the change in the ratio of absorbances per cell in each well. The change in the ratio of absorbances per cell for cells in modules with initial diameters of 1000, 750 and 500 μm were 1.8±0.5×10^−4^, 1.3±0.1×10^−4^, and 0.8 ± 0.1×10^−4^ respectively; these numbers indicate that the cells were metabolically active in all sizes of modules.

Trypan Blue is a dye that is excluded from living cells with intact membranes—dead cells stain blue, and live cells remain colorless [27]. To verify that large numbers of cells do not die in the modules, we digested the modules with a solution of dispase and trypsin to retrieve the encapsulated cells, and then performed a Trypan Blue test on these recovered cells. We counted the number of dead cells in the modules. No cells retrieved from modules after seven days in culture stained positively with Trypan Blue. This observation indicates that <1% of the cells in the modules were dead, and >99% were alive, after seven days in culture. Although we focused here on a one week culture period, culture in modules for longer periods is possible and has been reported previously in larger modules [24].

**Figure 2:**
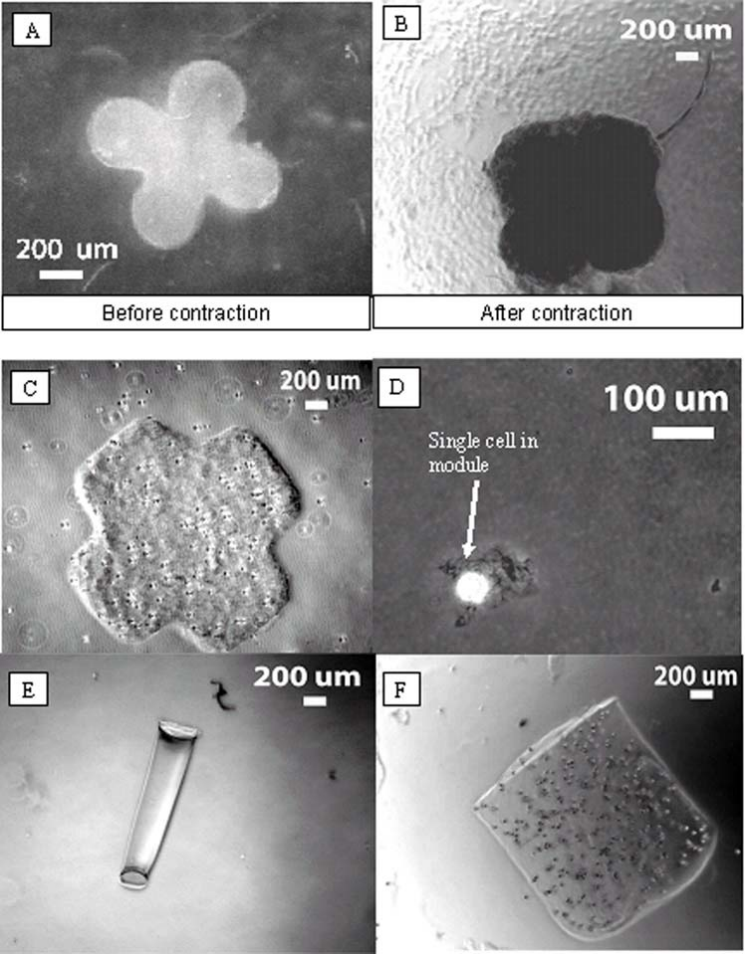
Light-microscopy images of modules fabricated in various shapes using various materials. A. Clover-shaped module fabricated from collagen, immediately after fabrication. Note that the magnification differs from that of other images. B. Clover-shaped module fabricated from collagen, 6 days after fabrication. Note scale bar. Significant (approx. 50%) contraction of the module occurred during 6 days in culture. C. Cross module fabricated from collagen, immediately after fabrication. D. Square cross-section module (40-μm wide) fabricated from collagen containing one cell only (bright spot in image), immediately after fabrication. E. Side view of square cross-section module (200-μm wide) fabricated from 2% agarose gel (containing no cells), immediately after fabrication. F. Cylindrical module (1 mm in diameter) fabricated from Matrigel™, immediately after fabrication.

**Figure 3:**
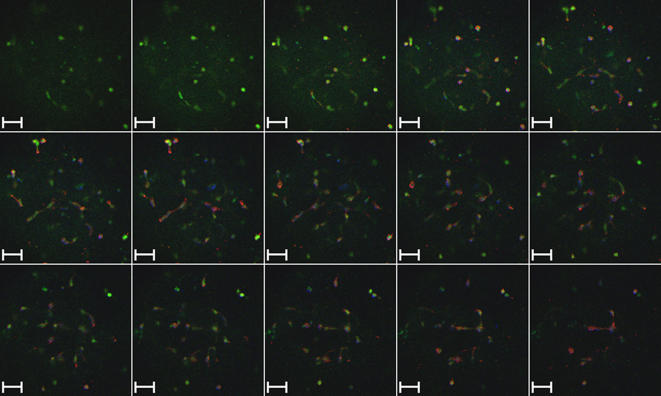
Confocal microscopy through a module after 2 days in culture. Series of images at increasing depth in a 500-μm wide module, 2 days after fabrication. The series of images indicates that cells are evenly distributed throughout the module. The scale bar for each image is 50-μm wide. DAPI (blue) indicates cell nuclei, CFSE (Green) indicates cell cytoplasm where esterase activity is present, and phalloidin (red) indicates actin filaments.

Light microscopy images suggested that cells were distributed evenly throughout a module after fabrication (Figure 2B, 2C and 2F), as long as the cells were evenly distributed in the collagen gel initially during fabrication of the modules. Mixing cellular aggregates instead of individual cells into the collagen during fabrication resulted in modules with uneven cell distributions, which may find use in some applications. Confocal microscopy was used to demonstrate even distributions of cells in modules formed using a well mixed suspension of cells. Figure 3 shows the even distribution of cells in successive layers, from the surface of the module to approximately 20 μm into the module. The focal length of the microscope was sufficient to enable visualization of the module to a depth equivalent to the thickness of approximately three cells. Beyond this depth, the laser did not penetrate the dense tissue sufficiently to produce a detectable signal.

We quantified the density of cells seven days after fabrication in cylindrical modules with three different initial diameters (1000, 750 and 500 μm). We determined the number of cells in the modules by digesting samples of 50 modules in a solution of dispase and trypsin (see Materials and Methods), followed by manually counting the released cells with a hemocytometer. The density of cells in the modules was calculated by dividing these counts by the average volume of a module after seven days in culture (determined from the module diameters and lengths as measured from images obtained by light microscopy—see below). Table 1 contains values of cell density for modules of different size. The density of cells ranged from 1.7×10^7^ to 2.3×10^8^ cells/cm^3^ as the size of the modules varied: smaller modules appeared to enable higher densities of cells than did larger modules (see Table 1). Cells may grow to higher densities in small modules than in larger modules because transport of nutrients to and from the center of a module is limited by diffusion in modules above a certain size. Making modules with diameters below 200 μm by the method presented here allows cells to proliferate in those modules to densities close to those found in tissues *in vivo* (10^8^ to 10^9^ cell/cm^3^).

**Table 1:** Characterization of cylindrical modules over time: Module length, diameter and cell density characterized immediately and 5 days after fabrication (mean±Standard error of the mean is indicated).

	1000 μm diameter modules	750 μm diameter modules (batch 1)	500 μm diameter modules (batch 1)
Mold diameter (μm)	1000	750	500
Mold length (μm)	800	500	500
Diameter measured immediately after Fabrication (μm)	1750±12 (n = 69)	890±10 (n = 75)	600±7 (n = 71)
Length measured immediately after Fabrication (μm)	925±75 (n = 6)	595±20 (n = 25)	540±19 (n = 18)
Diameter measured after day 7 (μm)	565±11 (n = 96)	280±7 (n = 66)	170±6 (n = 60)
Length measured after day 7 (μm)	460±11 (n = 70)	210±6 (n = 41)	265±12 (n = 32)
Volume of 1 module immediately after fabrication (cm^3^)	22×10^−4^	37×10^−5^	16×10^−5^
Volume of 1 module immediately after 5 days in culture (cm^3^)	12×10 ^−5^	13×10^−6^	61×10^−7^
Cell density immediately after fabrication based on seeding density (cells/cm^3^)	2×10^6^	2×10^6^	2×10^6^
Cells density in modules after 7 days (cells/cm^3^) *	17±3×10^6^	84±5×10^6^	23±1×10^7^

*Cell density in modules at day 5 was calculated by manually counting the number of cells per module and then dividing that number by the volume of one module calculated using the dimension measurements given in the table.

### Range and reproducibility of module dimensions

As 3T3 cells grow inside of the collagen, they attach to and pull on the collagen fibers of the gel to produce alignment of the collagen fibers and contraction of the gel [28]–[31]. Figure 2A – B shows the contraction of a clover-leaf shaped module during six days of growth in culture medium. The modules contracted to ∼4% of their original volume by the seventh day in culture (calculated by 100×V_f_/V_i_, See Supplemental Figure S2 for more information about the contraction of modules with circular cross-sections.) Contracted modules were easier to handle and less easily damaged by pipetting than were those before contraction, due to the increased strength of aligned collagen fibers. Contraction of the modules was not generally isotropic, and resulted in a change in the module aspect ratio (see Supplemental information). Contraction also caused some distortion in the shapes of modules: over time, indentations and corners of the modules tended to became rounded (Figure 2A–B). We expect that the extent of the module contraction is dependent on the rigidity of the matrix, the adhesiveness of cells to the matrix, the density of cells in the modules, and the type of cells encapsulated in the modules. For example, 3T3 cells produced significantly greater contraction of the modules than did HepG2 cells.

To demonstrate that it was possible to fabricate modules reproducibly in a broad range of sizes by this method, we encapsulated 3T3 cells in modules with lateral dimensions ranging from 40 μm (Figure 2D) to 1000 μm (Figure 2F). We used phase-contrast microscopy and the ImageJ (NIH freeware) image-processing program to collect statistics on batches of 50 modules at two times after fabrication. Each batch consisted of modules made exclusively from membranes containing holes 500, 750, or 1000 μm in diameter. Measurements confirmed that the length and diameter of the modules in any single batch were highly reproducible, both before and after contraction, to a standard error within 5% of the mean for that batch. The length and diameter of modules fabricated from identical membranes in any batch was reproducible to a standard error within 10% of the mean for those batches (see Table 1 and Supplemental Figure S3 for details).

### Versatility of the technique used to form modules

**Figure 4:**
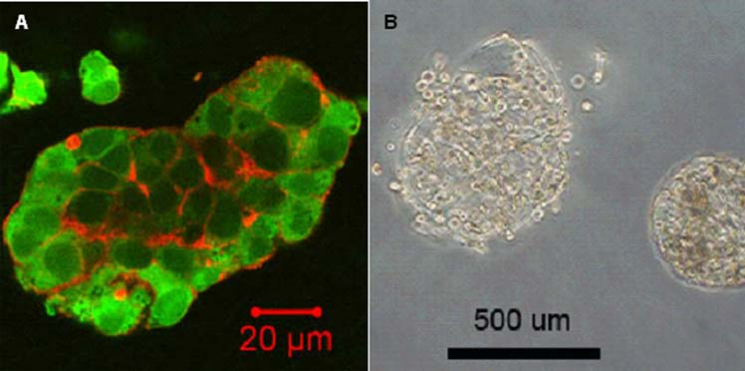
HepG2 cells and primary cardiomyocytes cultured in collagen modules. A. Confocal microscopy image of HepG2 cells in a 1-mm wide collagen module, cultured for 17 hours. DAPI (blue) indicates cell nuclei, and phalloidin (red) indicates actin. B. Light microscopy image of rat cardiomyocytes encapsulated in a 500-μm wide collagen module, cultured for 24 hours.

To demonstrate the versatility of our method, we fabricated modules from three different polymers (collagen, Matrigel™, and agarose) and in a variety of simple shapes (circles, squares, crosses, and circular crosses). Figure 2 contains light-micrographs of modules made from different gels with differently shaped cross-sections. To show the generality of our method, we encapsulated three different cell types: 3T3 fibroblasts (mouse), HepG2 cells (human, Fig. 4A), and primary rat cardiomyocytes (Fig. 4B). We focused mainly on 3T3 cells because they contract the collagen gel as they proliferate (unlike some other cell lines such as HepG2), and because there is extensive literature on fibroblasts in collagen gels [28]–[31].

We fabricated modules from type I Vitrogen™ collagen, because it is a commercially available material, commonly used for applications in tissue engineering and cell biology. Unlike some other types of collagen, Vitrogen™ collagen is free of endotoxin (<0.1 endotoxin units reported by manufacturer), which can change the behavior of some cell types. Since collagen is a major component of the extracellular matrix, it is an appropriate material to use in generating a “tissue-like” environment. We also used Matrigel™ as a material for modules, since it is also a widely used gel in tissue engineering and cell biology. We fabricated modules from agarose to demonstrate that rigid gels are also compatible with the method.

We fabricated modules with two different classes of shapes: (i) square and clover-leaf cross-sections, to demonstrate that our technique can generate modules with sharp corners and with various shapes; and (ii) circular cross-sections to allow easy comparison of our technique to the original process of McGuigan and Sefton, which also produced cylindrical modules [23], [24]. The ability to fabricate modules with different shapes may be useful for two reasons: (i) Encapsulating cells of different types in modules of different shapes or sizes allows discrimination by eye, gravity, or (in principle) velocity in a gentle flow. Separating mixtures of modules may be useful after co-culture experiments, for example. (ii) The shape of the modules controls how the modules pack in an aggregate, and thus controls the architecture of the network of pores that permeate the aggregate. When the modules are packed into a porous aggregate to form modular tissue, the cell-culture medium must continuously flow through the interconnected channels that permeate the aggregate to keep all the cells alive. The flow of liquid through these porous channels generates a shear stress on the channel walls, and hence the on cells that cover those walls. The magnitude of this stress depends on the architecture of the pores; the ability to control the shapes of modules, therefore, also provides control over the shear stress on the cells that line the channel walls.

### Comparison of cell morphology and function in 2D versus 3D

**Figure 5:**
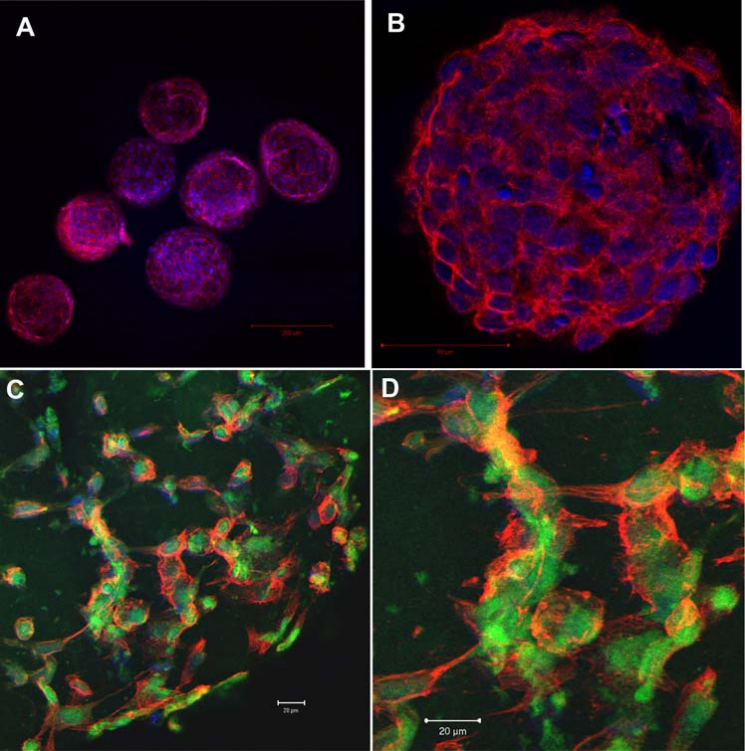
Confocal microscopy of modules after 7 days in culture. A. Low Magnification image of 3T3 cells in 500-μm wide collagen modules cultured for 7 days. DAPI (blue) indicates cell nuclei, and phalloidin (red) indicates actin. B. 3T3 cells in a 100-μm wide collagen module cultured for 7 days. DAPI (blue) indicates cell nuclei, and phalloidin (red) indicates actin. C. 3T3 cells in a 500-μm wide collagen module cultured for 15 days. DAPI (blue) indicates cell nuclei, CFSE (Green) indicates cell cytoplasm where esterase activity is present, and phalloidin (red) indicates actin filaments. D. Magnified section of Figure 5C.

The morphology of fibroblasts in the 3D matrix of the module differs from that of fibroblasts on a 2D surface. We used laser-scanning confocal microscopy to visualize interactions among cells grown in 3D at tissue-like densities (>10^7^ cells/cm^3^). Cells were stained with carboxyfluorescein diacetate succinimidyl ester (CFDA SE) to visualize their cytoplasm, DAPI to visualize their nuclei, and phalloidin to visualize the actin distribution within the cells. Figure 5 shows confocal images of 3T3 cells in modules seeded at high (Fig 5A and 5B) and low (Fig 5C and 5D) density of cells after one or two weeks in culture. At high densities of cells (Fig 5A and 5B), the fibroblasts packed tightly and exhibited a rounded, compact morphology, in contrast to the elongated spindle-like morphology characteristic of fibroblasts on a rigid 2D surface (see Supplemental Information Figure S4 for images of fibroblasts in 2D). At lower densities of cells (Fig 5C and 5D), individual fibroblasts clearly exhibited spherical bodies with cell-cell cytoskeletal extensions in all directions. Both actin filaments and cytoskeletal extensions were observed extending from one cell towards neighboring cells within the gel (Fig 5C and 5D) in all three dimensions. These results suggest that cells grown in 3D differ from cells grown in 2D in both the in morphology of individual cells, and in the extent of interactions among cells, since interactions are possible in all three dimensions. The modular system offers a convenient method for observing cell-cell interactions in 3D over a range of cell densities.

In addition to observing differences in cell morphology between 2D and 3D culture, we also wanted to use a system based on modules to highlight differences between cellular functions in 2D and 3D. Using ELISA, we quantified rates of albumin secretion from human HepG2 liver cells in 3D modules and on 2D rigid plastic. The rate of albumin secretion from HepG2 cells in modules was 0.465±0.041 pg mL^−1^ cell^−1^ h^−1^, but was only 0.114±0.004 pg mL^−1^ cell^−1^ h^−1^ from HepG2 cells cultured on 2D tissue culture treated plastic. Albumin secretion in 3D was significantly higher than in 2D (Student t-test p = 2.7×10^−4^); this finding is consistent with previous observations [32].

## Discussion

Besides their use in clinical applications, engineered tissues provide highly controllable and versatile systems for studying 3D cellular phenomena. Methods for fabricating and assembling engineered tissues however, are non-standardized, often involve specialized equipment [13], [25], and are not likely to be widely adopted by the biology community. In this paper, we adapt one particular tissue engineering approach—modular tissue engineering—to make an accessible tool for studying cells behavior in 3D. By using 3D modules with dimensions below 200 μm, investigators can generate densities of cells near those of native tissue. The modular strategy is simple and reproducible, compatible with commonly used biological tools such as microscopy and high-throughput technology, and the modular techniques allow significantly more control over the types of cells and extra-cellular matrixes in the microenvironment than do methods based on tissue explants or 3D cellular aggregates.

The most significant new capability of the technique we describe is the encapsulation of cells into gel modules whose small size allows nutrients to diffuse to all cells in the module. Removing limits to growth imposed by diffusion enables the cells to proliferate to tissue-like densities (10^8^ – 10^9^ cells/cm^3^). A high density of cells allows simulation of a realistic tissue environment for dense tissues with high metabolic rates, such as liver. Colton *et al.* have calculated the maximum non-anoxic dimensions for slab, cylindrical and spherical geometries using the appropriate Thiele modulus equations based on zero-order kinetics [18]. For an oxygen uptake rate equal to 2.5×10^8^ mol/cm^3^ s (a typical value) and an oxygen partial pressure of 40 mm of Hg, the maximum characteristic lengths (diameter) to avoid anoxic core formation are 128 μm, 182 μm and 224 μm for a slab, cylinder and sphere respectively [18]. If oxygen is the principle limiting factor on growth of cells (and therefore, on density of cells) in any 3D mass of cells, then the growth of cells in fabricated modules with diameters smaller than ∼200 μm will not be limited by transport of nutrient, and cells will thus proliferate to the desired density of cells (10^8^ – 10^9^ cells/cm^3^). We observed modules at these densities (see Table 1), and cells remained alive at high cell densities over at least seven days.

Interestingly, no dead cells were observed in modules with diameters *larger* than 200 μm, either (see Trypan Blue assay above). We also observed that initially smaller modules reached higher densities of cells after one week in culture than did larger ones. A possible explanation of these observations is that in large modules limitations on diffusion of oxygen to the center may allow the cells only to reach a density that enables all cells to remain viable; therefore, the types of cells that we used do not overextend themselves and create a hypoxic environment, at least over a period of one week. Further experiments are necessary to characterize (i) cell growth rates over time in modules of different sizes, and (ii) the cellular control mechanisms that regulate cell growth in the modules. A system based on modules in a high-throughput screen may serve to identify genes that regulate cell growth in 3D.

Another important feature of our method is the reproducibility and control (over module dimensions and shape) provided by lithographic techniques [5], combined with accessibility to biologists who do not have access to specialized equipment. Photolithography is a standard tool in electronics laboratories, but access to clean rooms and lithographic tools is often limited. Soft lithography provides an alternative method of microfabrication that is capable of generating comparable features as small as ∼1 μm in width [5], and other methods exist to fabricate features ∼200-μm wide without any use of a cleanroom at all [33]. In the method described here, transparency masks define the lateral dimension of the posts, which define the membrane holes that mold the modules. The masks are commercially available by custom order for less than $50. Generating variously shaped features in the transparency mask (and, therefore, in the final membrane used to generate the modules) is trivial, and enables the fabrication of modules with a wide variety of sizes and cross-sections. The fabrication of modules by photolithography, soft lithography, and related techniques is highly reproducible and yields far more modules than the original technique of McGuigan and Sefton [23], [24]. The ability to produce large quantities of indistinguishable modules will be critical to utilize modules in high-throughput screens and other applications where minimal variability between samples is important.

There are a number of other reported methods to encapsulate cells in polymer gels. McGuigan and Sefton filled flexible polyethylene tubing with collagen gel and cut the gel-filled tubing into short pieces [23], [24]. Vortexing the cut tubing separated the cylindrical gel core (the module) from the tubing. That method yielded cylindrical modules with diameters greater than 500 μm, required a custom-made cutter for the tubing, and took great deal of time. Recently attempts have been made to micromold cell-containing gels in chambers made from poly(dimethyl siloxane) by soft lithography [34]–[37], but none has enabled the generation of free-floating cell-containing modules fabricated from fragile gels such as collagen, or Matrigel™. None of these other methods allows the fabrication of sub-100 μm modules (which enable cells to grow at high cell densities), or the encapsulation of single cells (except in large amounts of excess matrix). Techniques for forming free-floating modules using *in situ* photolithography [13], [25] allow the production of free-floating modules, but only those with dimensions over 500 μm have been reported, and this alternative method requires specialized equipment and the use of photoreactive polymers to encapsulate the cells.

The method presented in this paper provides several critical improvements over these existing methods to encapsulate cells in gels: (i) smaller modules (and thus higher densities of cells) with a variety of shapes are possible. (ii) Modules made from both stiff gels like agarose and from common fragile biopolymers, such as collagen and Matrigel™ are possible. This flexibility in choice of material is important in a system for culturing cells, since different types of cells have different requirements to remain alive. Since each type of module is prepared separately, this method also may allow the assembly of culture systems that combine modules made from different materials, and that contain cells with different culture requirements. (iii) No need for specialized equipment, such as apparatus for dieletrophoresis or *in situ* photopolymerization, [13], [25]; the modular method is therefore more accessible to the biology community. (iv) No need for photopolymerization, which can damage cells, to form the modules.

Using the modular system, we investigated differences in the morphology and function of cells in 2D versus 3D environments. Confocal analysis of cells within the modules yielded detailed images of cell-cell interactions within the collagen gel. Inspection reveals obvious differences in morphology between 3T3 cells cultured in 3D and those cultured in 2D. The 100-μm modules were small enough (*i.e.,*the penetration into the gel matrix by the focal plane of the microscope was sufficient) to allow complete interrogation of every cell when using two photon laser (penetration depth around 150 μm), but only the first three layer of cells in the module were visible when using a conventional laser. By varying (i) the initial density of cells at the time of seeding and (ii) the time after seeding at which the cells were imaged, investigators can probe the behavior of cells in different microenvironments, with different cell densities, or with different extents of module contraction (and, presumably, different degrees of remodeling the collagen matrix). Mixing multiple cell types and multiple extracellular matrix components allows the generation of even more complex microenvironments within the modules. Furthermore, since the modules easily transfer from one container to another by using a pipette, they provide a convenient platform, in combination with live-imaging confocal analysis, for monitoring module morphogenesis and cell re-organization over time. In the future, other imaging techniques will be useful to probe more detailed aspects of cell morphology in the modules, and to compare these features to those seen in native tissue to establish how well the module environment represents native tissue. For example, electron microscopy could be used to visualize adhesion zones and other signs of cell differentiation, and could help to show matrix degradation and *de novo* synthesis, to better understand cellular remodeling of the matrix.

In addition to observing the morphology and organization of cells in 3D we wanted to quantify some aspects of cellular function in the 3D modules and highlight the differences between 3D and 2D. Furthermore, we wished to focus on readouts of cellular function that could be useful for studying cell-cell interactions in tissues, and in drug metabolism. We therefore focused on albumin secretion in the hepatocyte cell line HepG2. Consistent with previous reports, albumin secretion rates were increased in cells cultured in 3D gels [32]. It would be interesting in the future to perform similar analysis on modules containing mixtures of hepatocytes and nonparenchymal cells such as fibroblasts, either in the same or in separate modules, since nonparenchymal cells are known to affect albumin production rates in 2D systems [38]. In an analogy to a reported 2D system [39], the modular system allows co-culture of both cell types in 3D with either direct (both cell types in the same module) or indirect (each cell type in separate modules) contact between the two cell types, which would be much more difficult to achieve using cellular aggregates. The modules also offer a convenient platform for conducting high-throughput screens to establish the genes critical to the interaction between these two different cell types in 3D.

By increasing the accessibility of modular tissue engineering, we hope to enable its use in applications outside of tissue engineering, and to provide a simple method for biological investigations of the behavior of cells in 3D at high density. For example, for drug screening applications, the module system offers two unique opportunities over cellular aggregates: (i) it is possible to screen/test drugs on cells that do not readily form spheroids, such as cardiomyocytes; and (ii) it is possible to incorporate small particles, such as drug delivery microspheres, into the module matrix during fabrication and hence study the response of cells to drug delivery systems over time in 3D. To use this system for drug screening or testing, however, it will be important to use primary cells. We believe that the modular approach, combined with imaging techniques and other analytic tools such as ELISA, offers a powerful system for studying 3D biology in a number of different contexts.

## Materials and Methods

### Cell culture

The NIH 3T3 fibroblast cell line (American Type Culture Collection, Rockville, MD) was cultured in DMEM culture medium with L-glutamine (Invitrogen, Carlsbad, CA) supplemented with 10% fetal calf serum (Sigma-Aldridge) and 2% penicillin and streptomycin (Invitrogen). The human HepG2 cell line (American Type Culture Collection, Rockville, MD) was cultured in similar medium supplemented with 5% fetal calf serum. Primary neonatal rat ventricular cardiomyocytes were isolated from 2-day old neonatal Sprague-Dawley rats using methods described elsewhere [40].

### Membrane fabrication and cleaning

We used soft lithography to fabricate the membranes [5]. The membranes were open on both faces. Soft lithography combines the standard techniques of photolithography with moldable polymers to replicate structures as small as a few nanometers. Fabrication of the membrane required three steps: (i) photolithography to generate an array of permanent posts (25 – 800 μm high, 40 – 1000 μm wide) on a flat silicon substrate; (ii) spin-coating a layer of PDMS around the posts and peeling it away from the posts to yield a thin membrane with an array of holes; (iii) cleaning the membrane and removing air bubbles from the holes so that gel can enter.

Fabrication of posts by photolithography requires a transparency mask (from Pageworks.com, Cambridge, MA or Outputcity.com, Bandon, OR) to define the desired dimensions. We designed an array of transparent features against a black background by using a computer drafting program (Macromedia Freehand 10 or CleWin). The length and width of the transparent regions of the mask determined the length and width of the posts. We followed the manufacturer's instructions for spin-coating, baking, exposing, and developing the resist. We used spin-coating of SU-8 50 photoresist (Microchem Corp., Newton, MA) on a clean silicon wafer (d = 7.5 cm) to obtain a smooth layer of photoresist with even thickness. The thickness of the photoresist determined the height of the posts, and therefore also defined the maximum depth of holes in a membrane molded around those posts (see Figure 1). Exposing SU-8 50 photoresist to ultraviolet light (AB-M mask aligner, 365 nm light) rendered the photoresist insoluble; the mask allowed us to expose only certain regions (the posts). After developing in solvent (propylene glycol methyl ether acetate), all unexposed regions of the photoresist dissolved to give a silicon wafer that bore an array of three-dimensional features with the desired dimensions. To render the surface of the wafer and features “non-stick,” we placed the developed wafer in a dessicator at 20 Torr with 3 drops of 1,1,2,2-tetrahyrooctyl-tridecafluorotrichlorosilane (United Chemical Technologies) for 3 hours. The silane vaporized at these conditions, and gradually reacted with the surface of the wafer and posts to give a self-assembled monolayer of fluoronated, Teflon-like molecules.

To form the membranes, we mixed Dupont Sylgard 184 poly(dimethyl siloxane) polymer base with the liquid catalyst (10∶1 by mass) and stirred vigorously according to the manufacturer's instructions. We degassed the mixture in a dessicator at 20 Torr for 45 minutes to remove the air bubbles. We spin-casted degassed PDMS for 20 seconds over the posts. Spin-casting at higher speeds yielded thinner membranes (800 μm, 250 RPM; 500 μm, 500 rpm; 200 μm, 1000 rpm; 50 μm, 2230 rpm). To ensure that no PDMS remained on top of the posts, we gently swept a flat, 1-mm thick slab of solid PDMS over the posts. The PDMS cured at 70 °C for 1 hour.

Generally, the surface of PDMS is hydrophobic. Aqueous solutions of biopolymers are better able to infiltrate holes in PDMS membranes when the PDMS is hydrophilic. We oxidized the membranes in air plasma (SPI Plasma Prep II) for 1 minute to render the surfaces hydrophilic, and stored them under Millipore water to preserve their hydrophilicity [41]. We kept the submerged membranes at 20 Torr to remove air bubbles from the holes. After use, membranes were rinsed with a 2% solution of Micro-90 detergent (International Product Corporation), and then sonicated in ethanol for 20 minutes. We re-oxidized the membranes after cleaning, and returned them to Millipore water. Membranes were sterilized in water by autoclaving.

### Module fabrication

NIH 3T3 cells, HepG2 cells, or primary cardiomyocytes (10^6^ – 10^7^ cells per mL of collagen-containing solution) at room temperature were mixed with a solution of collagen (3 mg/mL, Inamed Biomaterials, Fremont, CA) containing 10× minimum essential medium (125 μL per mL collagen, Gibco/ Invitrogen, USA) and pH neutralized with 1 N sodium bicarbonate. The gel-cell mix was infiltrated into the holes by submerging the membrane in approximately 3 mL of the fluid in a 5-cm wide Petri dish (see Figure 1). The membrane was suspended on the rim of a Petri dish in an incubator and the gel was allowed to solidify at 37 °C for 45 to 60 minutes. Modules were then removed from the holes by agitating the membranes with tweezers while submerged in a Petri dish containing cell culture medium.

### Module characterization

#### Cell viability and cell density

The viability of cells in modules, 7 days after the fabrication of the modules, was measured using the Alamar Blue assay (AB). Aliquots of 50 modules were collected in a pipette tip in 60 μL culture medium, and added to a 96 well plate (50 modules per well). A solution of resazurin (the Alamar Blue compound; 10% by volume) was added to each well containing the modules in medium, and the plate was incubated for 2 hours and 20 minutes at 37 °C. Samples (50 μL) of the culture medium containing resazurin were then removed from each well (the modules remained in the well) and added to a fresh 96 well plate (by transferring the colored supernatant into a fresh well while measuring absorbance, we ensured that there were no modules present in solution to cause inaccuracies in absorbance readings). The absorbance of each sample was read in a Molecular Devices SpectraMax (provided by the FAS Center for Systems Biology) plate reader at 570 nm and 600 nm. The reduction of resazurin, a measure of metabolic activity was calculated as per the manufacturer's instructions. The same modules were then digested by using solutions of dispase (BD Biosciences, 100 μL (∼5000 caseinolytic units), 45 mins at 37 °C) and trypsin (100 μL, additional 15 mins at 37 °C)). We pipetted modules mixed with dispase and trypsin through a 200 μL pipette tip 50 – 100 times to form a suspension of single cells. We mixed samples of this suspension with Trypan Blue and counted the number of live cells (colorless) and dead cells (blue). We recorded the cell counts for specific wells so we could calculate the change in ratio of absorbances per cell for each well. We averaged the results over 50 modules in each batch by dividing the average change in ratio of absorbances in 50 modules (calculated from absorbance readings of each well) by the number of cells in 50 modules (calculated from manual cell counts from each well)

### Module dimensions

Images of the modules were taken using an Leica dissection microscope on the day of fabrication and 7 days after fabrication. The dimensions of the modules were measured from the images and used to calculate the extent of contraction of the modules over time as the cells populated and remodeled the collagen gel.

### Confocal microscopy

Confocal images were collected on a Zeiss LSM 510 system (Zeiss, Maple Grove, MN) equipped with 488 nm, 543 nm, and 633 nm diode lasers and a tunable two-photon laser (Coherent Chameleon, Santa Clara, CA). Gels containing living cells were fixed with 3% formaldehyde (v/v) for 20 minutes at room temperature. They were stained using CFSE (Molecular Probes, Eugene, OR) at 10 μM concentration for 30 minutes at 37 °C, and were also stained with rhodamine-labeled phalloidin (Molecular Probes, Carlsbad, CA) and DAPI (Invitrogen, Carlsbad, CA) using the manufacturers instructions. The structures were pipetted onto poly-L-lysine coated slides (Fisher Scientific, Pittsburgh, PA), blotted with Kim-Wipes to remove fluid, mounted with Gel/Mount (BioMeda, Foster City, CA), and covered with a coverslip. Images were obtained using oil-immersion 25x, 40x, and 63x lenses. Laser and detector settings for the confocal microscope were manually adjusted to prevent saturation of pixels or excess photobleaching. Each pixel was excited for 1.6 μs by each laser. Images were obtained in the x-y plane (1024×1024 pixels) and multiple z-sections (of thickness equal to the optimal optical slice width as calculated by the Zeiss software) were combined to form a composite 3D image. Deconvolution of images was performed using LSM software (Zeiss, Maple Grove, MN). All parts of each image were processed identically within each dataset. Scattering by the collagen matrix and the cells themselves limited visualization into the module to a depth of around 150 μm when using two-photon laser, and to around 20 μm when using the conventional lasers.

### Quantification of albumin secretion rate

We placed either 20000 HepG2 cells, or 5 modules containing HepG2 cells (cell density 10^7^ cells/cm^3^ of collagen) into a well of a 96-well plate containing 200 μL of fresh culture medium for 17 or 24 hours. We then collected samples of the culture medium to measure albumin secretion by using a human albumin ELISA kit (Bethyl Laboratories, Inc., Montgomery, TX) according to the manufacturer's instructions.

## Supporting Information

Figure S1 Schematic diagram of modular tissue engineering strategy (adapted from ref 22).
(7.16 MB TIF)

Figure S2 Cylindrical modules of different sizes before and after shrinkage. Cylindrical modules fabricated in different sizes, imaged immediately and 5 days after fabrication. Significant shrinking of the module occurred as cells proliferated in the gel A. 1000-um diameter module, immediately after fabrication. B. 1000-um diameter module, 5 days after fabrication (not the same specific module as in A) C. 750-um diameter module, immediately after fabrication. D. 750-um diameter module, 5 days after fabrication (not the same specific module as in C) E. 500-um diameter module, immediately after fabrication. F. 500-um diameter module, 5 days after fabrication. (not the same specific module as in E).
(9.17 MB TIF)

Figure S3 Box plots showing variation of module dimensions within and among batches. Box plots display the within-batch and between-batch variation in measured A) diameters and B) lengths of the modules. Box plots are shown for batches of modules made from different molds and measured at zero and seven days after fabrication. The arrows connect the boxplot of a particular batch at day zero to the boxplot of that same batch of modules at day seven. The thick central line in the box represents the median, the ends of the box represent the first and third quartile of the data (values of measurements ranked at 25 and 75% respectively), the whiskers that extend from the box extend to the maximum and minimum measured values that lie within 1.5 times the inter-quartile range, and open circles and stars represent outliers and extreme outliers respectively. The box plots in Supplemental Figure S8 indicate the narrow size distribution of the contracted modules-small differences immediately after fabrication became almost undetectable after proliferation of cells and contraction of the gel. We note that the dimensions of modules measured immediately after fabrication and 3 hours later were consistently larger than the membrane holes from which they were molded; this change is probably due to swelling of the collagen by the liquid medium. If a particular module size is desired for an application, the size of the membrane mold must allow for swelling after fabrication.
(11.01 MB TIF)

Figure S4 Light-microscopy image of 3T3 fibroblasts cultured on a 2D surface.
(7.22 MB PNG)

## References

[1] Barralet JE, Wang L, Lawson M, Triffitt JT, Cooper PR (2005). Comparison of bone marrow cell growth on 2D and 3D alginate hydrogels.. J Mater Sci Mater Med.

[2] Liu H, Roy K (2005). Biomimetic three-dimensional cultures significantly increase hematopoietic differentiation efficacy of embryonic stem cells,. Tissue Eng.

[3] Weaver VM, Petersen OW, Wang F, Larabell CA, Briand P (1997). Reversion of the malignant phenotype of human breast cells in three-dimensional culture and in vivo by integrin blocking antibodies,. J Cell Biol.

[4] Li GN, Livi LL, Gourd CM, Deweerd ES, Hoffman-Kim D (2007). Genomic and morphological changes of neuroblastoma cells in response to three-dimensional matrices.. Tissue Eng.

[5] Xia Y, Whitesides MG (1998). Soft Lithography.. Angew Chem.

[6] Humphreys P, Jones S, Hendelman W (1996). Three-dimensional cultures of fetal mouse cerebral cortex in a collagen matrix.. J Neurosci Methods.

[7] Puri S, Hebrok M (2007). Dynamics of embryonic pancreas development using real-time imaging.. Dev Biol.

[8] Lim F, Sun AM (1980). Microencapsulated islets as bioartificial endocrine pancreas,. Science.

[9] Fukudaa J, Khademhosseini A, Yeoa Y, Yanga X, Yeha J (2006). Micromolding of photocrosslinkable chitosan hydrogel for spheroid microarray and co-cultures,. Biomaterials.

[10] Lazar A Peshwa MV, Wu FJ, Chi CM, Cerra FB (1995). Formation of porcine hepatocyte spheroids for use in a bioartificial liver,. Cell Transplant.

[11] Abu-Absi SF, Friend JR, Hansen LK, Hu WS (2002). Structural polarity and functional bile canaliculi in rat hepatocyte spheroids.. Exp Cell Res.

[12] Lazar A, Mann HJ, Remmel RP, Shatford RA, Cerra FB et al (1995). Extended liver-specific functions of porcine hepatocyte spheroids entrapped in collagen gel,. In Vitro Cell Dev Biol Anim.

[13] Albrecht DR, Tsang VL, Sah RL, Bhatia SN (2005). Photo- and electropatterning of hydrogel-encapsulated living cell arrays,. Lab on a Chip.

[14] Berthiaume F, Moghe PV, Toner M, Yarmush ML (1996). Effect of extracellular matrix topology on cell structure, function, and physiological responsiveness: hepatocytes cultured in a sandwich configuration,. FASEB J.

[15] Powers MJ, Janigian DM, Wack KE, Baker CS, Beer Stolz D (2002). Functional behavior of primary rat liver cells in a three-dimensional perfused microarray bioreactor,. Tissue Eng.

[16] Karp JM, Yeh J, Eng G, Fukuda J, Blumling J (2007). Controlling size, shape and homogeneity of embryoid bodies using poly(ethylene glycol) microwells.. Lab Chip.

[17] Soranzo C, Della Torre G, Ingrosso A (1986). Formation, growth and morphology of multicellular tumor spheroids from a human colon carcinoma cell line (LoVo), Tumori..

[18] Avgoustiniatos ES, Colton CK, Lanza R, Langer R, Chick W (1997). Design considerations in immunoisolation,. Principles of tissue engineering,.

[19] Nomi M, Atala A, Coppi PD, Soker S (2002). Principals of neovascularization for tissue engineering,. Mol Aspects Med.

[20] Choi NW, Cabodi M, Held B, Gleghorn JP, Bonassar LJ (2007). Microfluidic scaffolds for tissue engineering.. Nat Mater.

[21] Radisic M, Park H, Chen F, Salazar-Lazzaro JE, Wang Y (2006). Biomimetic approach to cardiac tissue engineering: oxygen carriers and channeled scaffolds,. Tissue Eng.

[22] McGuigan AP, Sefton MV (2006). Vascularized organoid engineered by modular assembly enables blood perfusion.. Proc Natl Acad Sci U.S.A..

[23] McGuigan AP, Leung B, Sefton MV (2007). Fabrication of a modular tissue-engineered organoid,. Nature Protocols.

[24] McGuigan AP, Sefton MV (2007). Design and fabrication of sub-mm sized modules containing encapsulated cells for modular tissue-engineering,. Tissue Eng.

[25] Liu Tsang V, Chen AA, Cho LM, Jadin KD, Sah RL (2007). Fabrication of 3D hepatic tissues by additive photopatterning of cellular hydrogels,. FASEB J.

[26] Ahmed SA, Gogal RM, Walsh JE (1994). A new rapid and simple non-radioactive assay to monitor and determine the proliferation of lymphocytes: an alternative to [3H]thymidine incorporation assay.. J Immunol Methods.

[27] Phillips HJ, Terryberry JE (1957). Counting actively metabolizing tissue cultured cells,. Exp Cell Res.

[28] Finesmith TH, Broadley KN, Davidson JM (1990). Fibroblasts from wounds of different stages of repair vary in their ability to contract a collagen gel in response to growth factors,. J Cell Physiol.

[29] Bell E, Ivarsson B, Merrill C (1979). Production of a tissue-like structure by contraction of collagen lattices by human fibroblasts of different proliferative potential in vitro,. Proc Natl Acad Sci U.S.A..

[30] Ehrlich HP, Rittenberg T (2000). Differences in the mechanism for high- versus moderate-density fibroblast-populated collagen lattice contraction.. J Cell Physiol.

[31] Kamamoto F, Paggiaro AO, Rodas A, Herson MR, Mathor MB (2003). A wound contraction experimental model for studying keloids and wound-healing modulators.. Artif Organs.

[32] Kono Y, Roberts EA (1996). Modulation of the expression of liver-specific functions in novel human hepatocyte lines cultured in a collagen gel sandwich configuration.. Biochem Biophys Res Commun.

[33] Cygan, ZT, Cabral JT, Beers K L, Amis EJ (2005). Microfluidic Platform for the Generation of Organic-Phase Microreactors,. Langmuir.

[34] Yeh J, Ling Y, Karp JM, Gantz J, Chandawarkar A (2006). Micromolding of shape-controlled, harvestable cell-laden hydrogels,. Biomaterials.

[35] Tan W, Desai TA (2005). Microscale multilayer cocultures for biomimetic blood vessels.. J Biomed Mater Res A.

[36] Tan W, Desai TA (2003). Microfluidic patterning of cellular biopolymer matrices for biomimetic 3-D structures,. Biomed Microdev.

[37] Tan W, Desai TA (2004). Layer-by-layer microfluidics for biomimetic three-dimensional structures,. Biomaterials.

[38] Bhatia SN, Balis UJ, Yarmush ML, Toner M (1999). Effect of cell-cell interactions in preservation of cellular phenotype: cocultivation of hepatocytes and nonparenchymal cells.. FASEB J.

[39] Hui EE, Bhatia SN (2007). Micromechanical control of cell-cell interactions,. Proc Natl Acad Sci U S A.

[40] Feinberg AW, Feigel A, Shevkoplyas SS, Sheehy S, Whitesides GM (2007). Muscular thin films for building actuators and powering devices,. Science.

[41] Morra M, Occhiello E, Garbassi F, Maestri M, Bianchi R (1990). The characterization of plasma-modified polydimethylsiloxane interfaces with media of different surface energy.. Clin Mater.

