# Correction: Cell Encapsulation in Sub-mm Sized Gel Modules Using Replica Molding

**DOI:** 10.1371/annotation/0a0b70a9-a6e0-4e60-b6bd-8ef894ed22cd

**Published:** 2008-07-11

**Authors:** Alison P. McGuigan, Derek A. Bruzewicz, Ana Glavan, Manish J. Butte, George M. Whitesides

The fourth author's name appears incorrectly in the author byline and in the citation. It should read: Manish J. Butte, and the citation should read: McGuigan AP, Bruzewicz DA, Glavan A, Butte MJ, Whitesides GM (2008) Cell Encapsulation in Sub-mm Sized Gel Modules Using Replica Molding. PLoS ONE 3(5): e2258. doi:10.1371/journal.pone.0002258

