# Supplementary figures and images for: Cell Encapsulation in Sub-mm Sized Gel Modules Using Replica Molding
Source: PLoS One. 2008 May 21;3(5):e2258. doi: 10.1371/journal.pone.0002258 (PMC2376064; doi:10.1371/journal.pone.0002258)

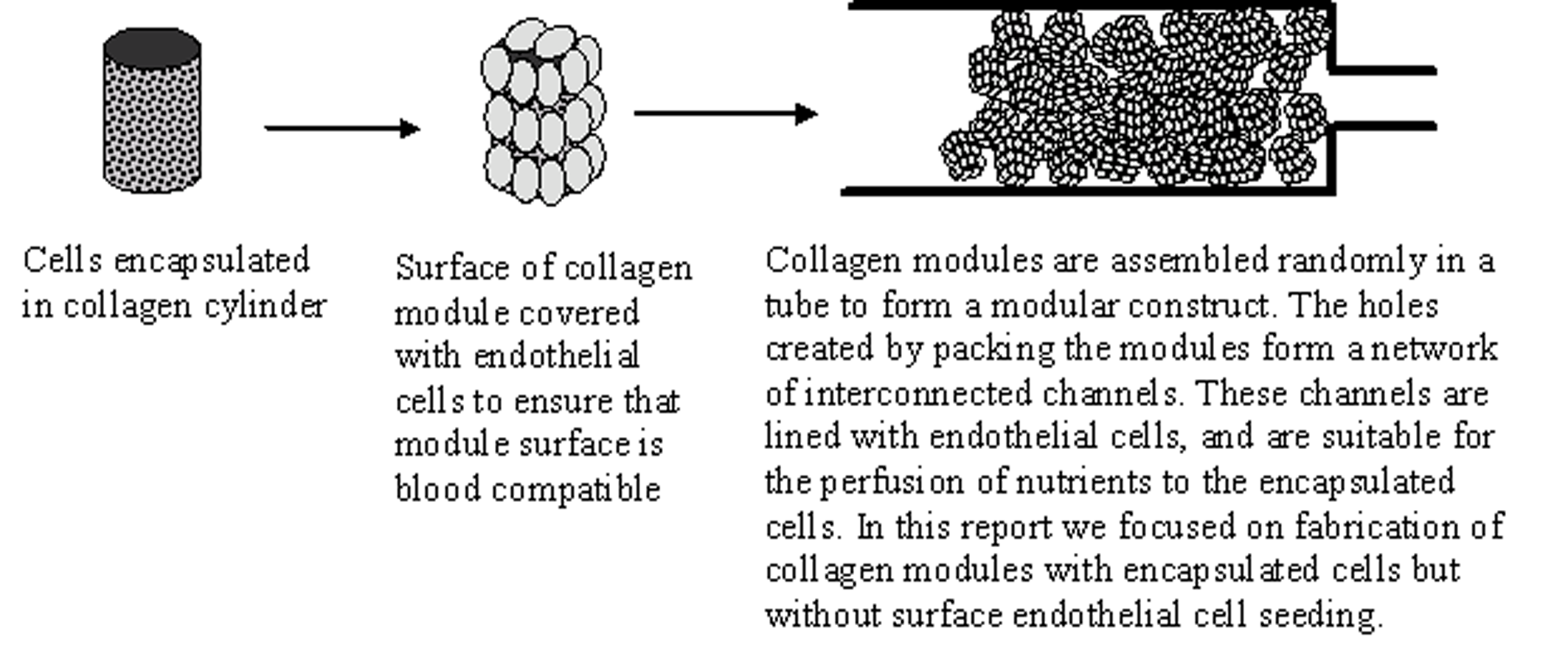

Supplement: Figure S1 — Schematic diagram of modular tissue engineering strategy (adapted from ref 22). (7.16 MB TIF) [file pone.0002258.s001.tif]

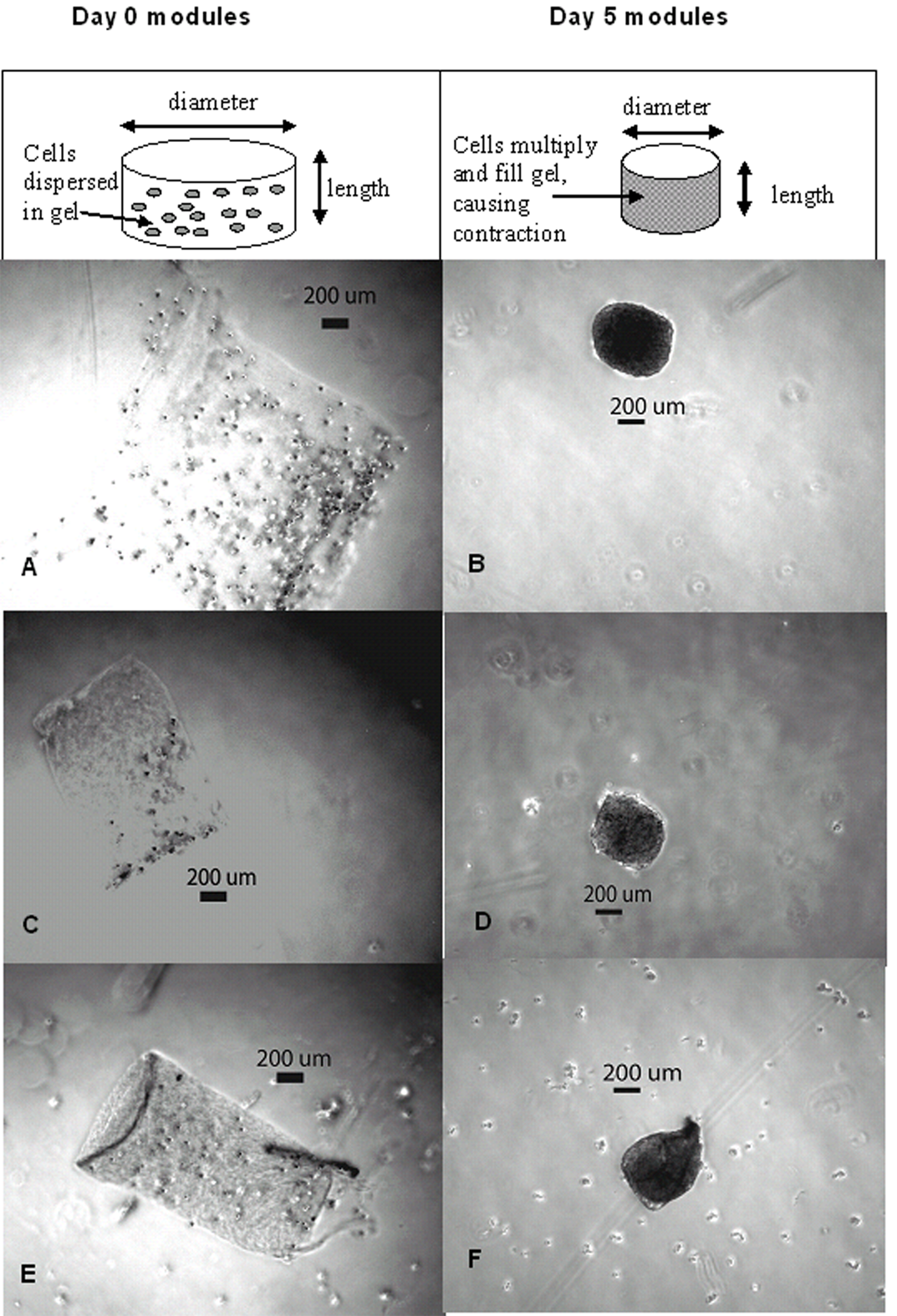

Supplement: Figure S2 — Cylindrical modules of different sizes before and after shrinkage. Cylindrical modules fabricated in different sizes, imaged immediately and 5 days after fabrication. Significant shrinking of the module occurred as cells proliferated in the gel A. 1000-um diameter module, immediately after fabrication. B. 1000-um diameter module, 5 days after fabrication (not the same specific module as in A) C. 750-um diameter module, immediately after fabrication. D. 750-um diameter module, 5 days after fabrication (not the same specific module as in C) E. 500-um diameter module, immediately after fabrication. F. 500-um diameter module, 5 days after fabrication. (not the same specific module as in E). (9.17 MB TIF) [file pone.0002258.s002.tif]

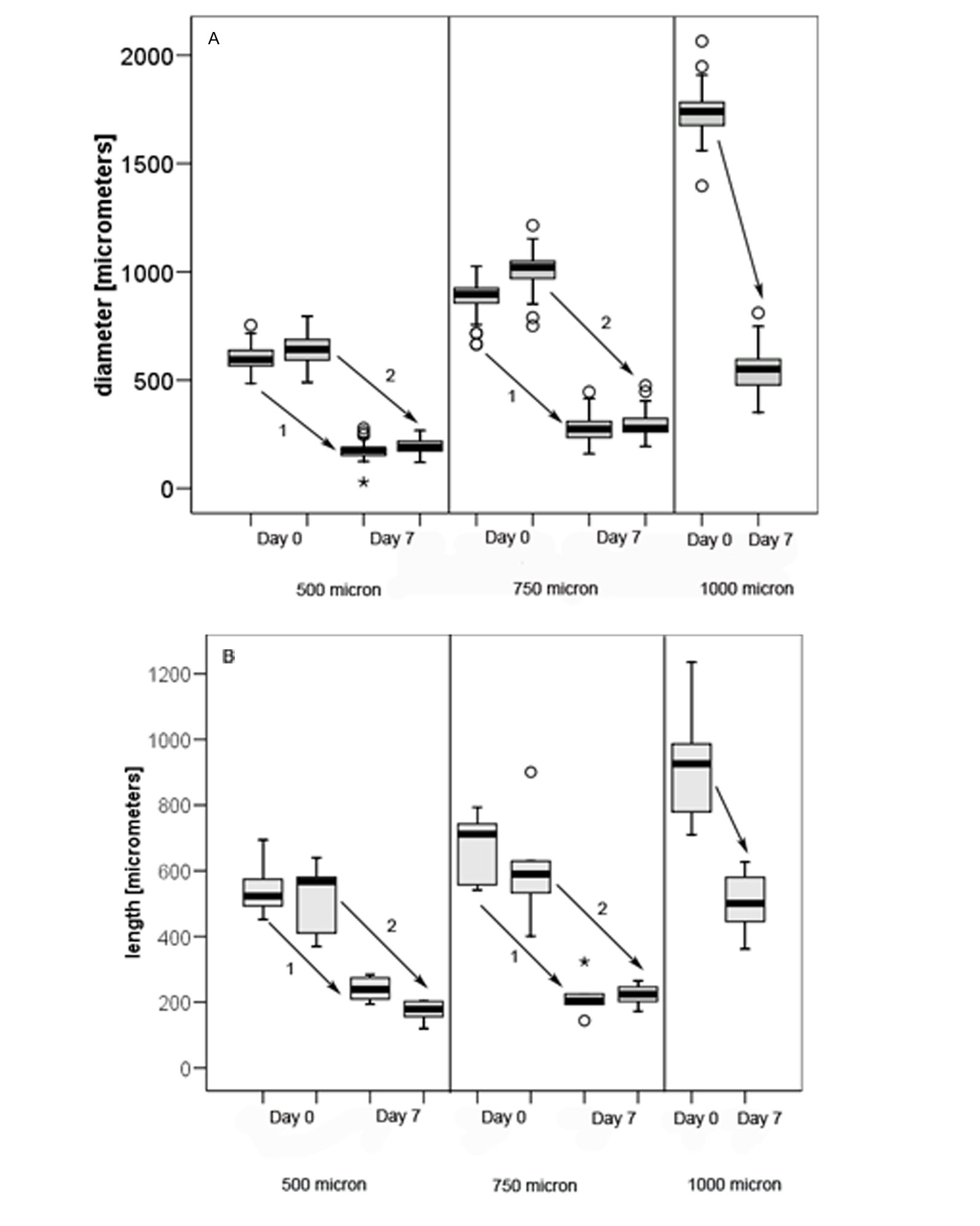

Supplement: Figure S3 — Box plots showing variation of module dimensions within and among batches. Box plots display the within-batch and between-batch variation in measured A) diameters and B) lengths of the modules. Box plots are shown for batches of modules made from different molds and measured at zero and seven days after fabrication. The arrows connect the boxplot of a particular batch at day zero to the boxplot of that same batch of modules at day seven. The thick central line in the box represents the median, the ends of the box represent the first and third quartile of the data (values of measurements ranked at 25 and 75% respectively), the whiskers that extend from the box extend to the maximum and minimum measured values that lie within 1.5 times the inter-quartile range, and open circles and stars represent outliers and extreme outliers respectively. The box plots in Supplemental Figure S8 indicate the narrow size distribution of the contracted modules-small differences immediately after fabrication became almost undetectable after proliferation of cells and contraction of the gel. We note that the dimensions of modules measured immediately after fabrication and 3 hours later were consistently larger than the membrane holes from which they were molded; this change is probably due to swelling of the collagen by the liquid medium. If a particular module size is desired for an application, the size of the membrane mold must allow for swelling after fabrication. (11.01 MB TIF) [file pone.0002258.s003.tif]

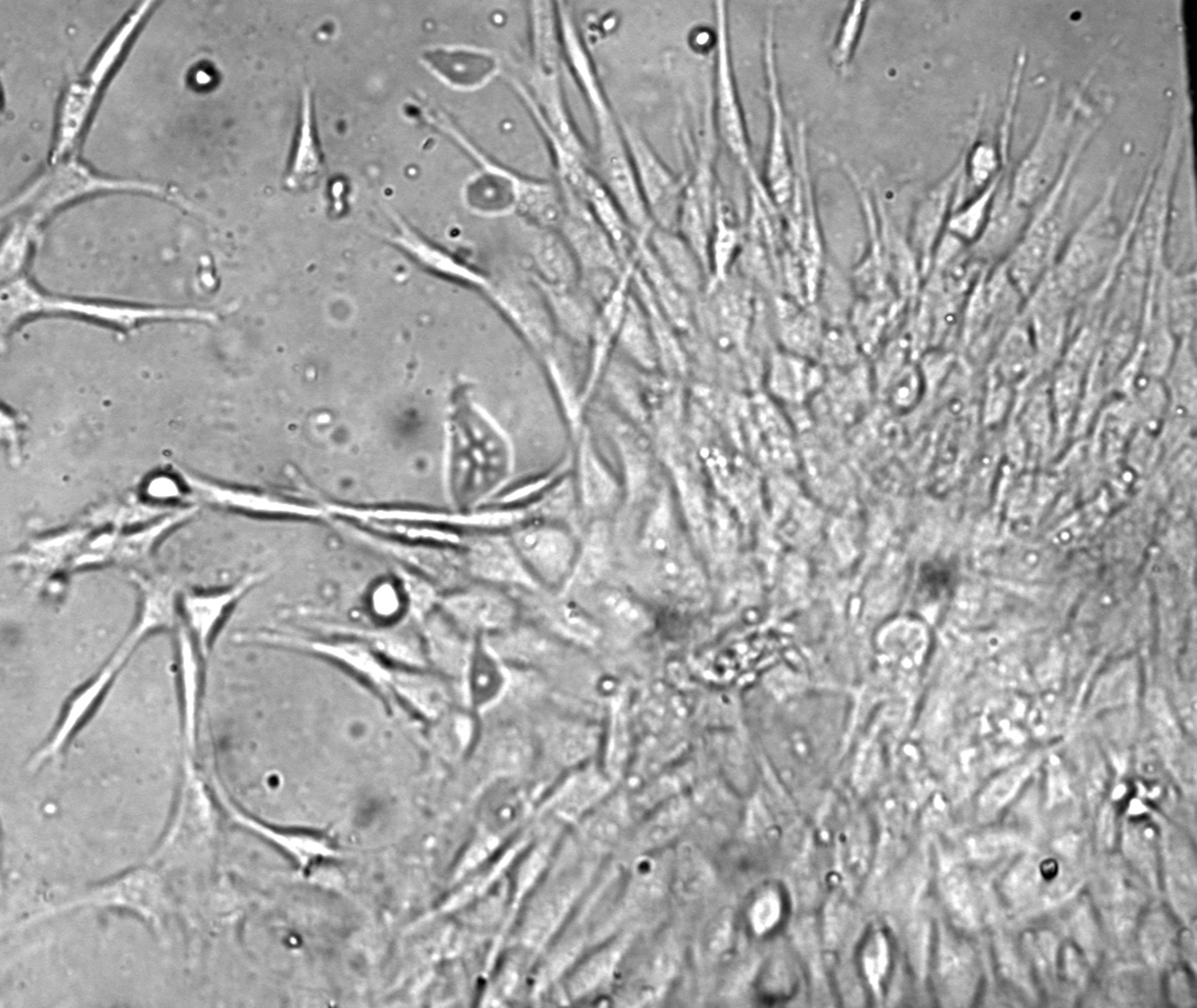

Supplement: Figure S4 — Light-microscopy image of 3T3 fibroblasts cultured on a 2D surface. (7.22 MB PNG) [file pone.0002258.s004.png]
